# Ontogenetic Variation and Sexual Dimorphism of Beaks among Four Cephalopod Species Based on Geometric Morphometrics

**DOI:** 10.3390/ani13040752

**Published:** 2023-02-19

**Authors:** Chao Wang, Zhou Fang

**Affiliations:** 1 College of Marine Sciences, Shanghai Ocean University, Shanghai 201306, China; 2 National Engineering Research Center for Oceanic Fisheries, Shanghai Ocean University, Shanghai 201306, China; 3 Key Laboratory of Sustainable Exploitation of Oceanic Fisheries Resources, Ministry of Education, Shanghai Ocean University, Shanghai 201306, China; 4 Key Laboratory of Oceanic Fisheries Exploration, Ministry of Agriculture and Rural Affairs, Shanghai 201306, China; 5 Scientific Observing and Experimental Station of Oceanic Fishery Resources, Ministry of Agriculture and Rural Affairs, Shanghai 201306, China

**Keywords:** cephalopods, beaks, ontogenetic variation, sexual dimorphism, geometric morphometrics, phenotypic plasticity

## Abstract

**Simple Summary:**

*Octopus minor*, *Uroteuthis edulis*, *Sepia esculenta* and *Sthenoteuthis oualaniensis* are important economic cephalopod species in coastal waters of China. They are very important role in marine ecosystems as relevant prey for large marine fish, and marine mammals are a critical component of the food chain. As the main feeding organ of cephalopods, the beak has a stable structure and is resistant to corrosion. However, the sexual dimorphism on the beak of the *O. minor*, *U. edulis* and *S. esculenta* and *Sthenoteuthis oualaniensis* at different ontogenetic stages is unknown, neither has it been determined whether variation of beak shape relates to changes in habitat environment and feeding preference at different ontogenetic stages. Using a geometric morphometrics approach, we found that the beaks of *O. minor*, *U. edulis*, *S. esculenta*, and *Sthenoteuthis oualaniensis* showed a pattern of variation, displaying sexual dimorphism and allometry between ontogenetic stages. Habitat may drive variation in beak shape. This study has furthered our understanding of the beak shape ontogenetic variation among the four species. We discuss the potential factors underlying the beak shape variation and provide a basis for the understanding of cephalopod phenotypic plasticity and its ecological significance.

**Abstract:**

Investigating the ontogenetic variation of biological individuals helps us to fully understand the characteristics of evolution. In order to explore the ontogenetic variation and sexual dimorphism of the beak shape in *Octopus minor*, *Uroteuthis edulis*, *Sepia esculenta* and *Sthenoteuthis oualaniensis* of the China’s coastal waters, the differences between immature and mature stages and the sex-linked differences in the beak shape and size were analyzed with geometric morphometrics methods in this study. The results of Procrustes analysis of variance, principal component analysis and multivariate regression showed that the shapes of the upper beaks of *O. minor*, *U. edulis* and *S. esculenta* differed significantly among various ontogenetic stages (*p* < 0.05). The shapes of the lower beaks of *U. edulis*, *S. esculenta* and *Sthenoteuthis oualaniensis* were also significantly different among various ontogenetic stages (*p* < 0.05). The results of thin-plate spline deformation grids showed that the beaks of the four cephalopod species presented different variation patterns. This study gives us basic beak geometry morphology information for *Octopus minor*, *Uroteuthis edulis*, *Sepia esculenta* and *Sthenoteuthis oualaniensis* present in China’s coastal waters. The ontogenetic differences in beak shape might be related to extrinsic factors (diet difference and intra and interspecific competition) in habitat.

## 1. Introduction

Biological individuals in nature often face different habitat environments which can drive phenological variation [1] and the evolution of organisms. Shape variations of biological individuals also reflect variation in their habitats [2]. In addition, the phenomenon of sexual dimorphism is also common in biological groups due to natural selection and gender selection. Sexual selection usually affects males through female choice and may also be caused by differences in lifestyle habits [3,4,5,6].

Two methods are generally used to explore ontogenetic and sexual dimorphism. First, based on traditional morphometrics where the linear distance difference among individuals is analyzed by multivariate statistical methods. However, the shape variation of biological individuals involves geometric information, which traditional morphometrics tend to ignore [7]. Secondly, geometric morphometrics (GM) was proposed in the 1990s, which addressed the problems of traditional morphometrics [8,9,10]. This method can quantify the geometric variation of organisms and accurately measure complex biological shapes. Therefore, it is widely used in morphological studies [11,12,13]. GM is the statistical analysis of the form based on Cartesian landmark coordinates [7,10,14]. InGM, by using a superimposition method, for instance, Procrustes, which is used to remove the effect of size, position and orientation of the landmark configurations, exclusively shape variables are obtained. Then, the Procrustes shape coordinates variation and influencing factors are studied by multivariate statistical methods, such as principal component analysis (PCA) and discrimination analysis (DA). GM is an effective method to evaluate shape variations at the intraspecific level as it allows the assessment of ontogenetic changes and sexual dimorphism [15], as has been evidenced for *Diplodus puntazzo* [16], *Munida rugosa* [17], *Lates niloticus* [1] and *Hysterocarpus traskii* [18]. Assessing ontogenetic changes and sexual dimorphism of organism structures can help distinguish taxa (e.g., species, subspecies and population) and understand their possible ecological role [18,19,20].

Cephalopoda contains around 850 extant species [21], which are widely distributed in the world. Cephalopods play an important role in marine ecosystems as relevant prey for large marine fish, and marine mammals are also a critical component of the food chain [22,23,24,25]. Phenotypic plasticity in response to environment variability is one of the main characteristics of cephalopods [26,27]. Storero et al. [26] showed the variability and plasticity of *Octopus tehuelchus* in response to the environment. Fang et al. [27] discussed the possible phenotypic plasticity of body and beak of *Ommastrephes bartramii* in different aspects. Therefore, exploring the life cycle of different cephalopods is prerequisite for sustainable exploitation and utilization of this resource.

As the main feeding organ of cephalopods, the beaks are located in the buccal mass and divided into the upper beak and the lower beak [15,28,29,30]. A beak is a hard tissue structure, which has a stable structure and is resistant to corrosion [28]. Therefore, it is widely used in the study of feeding ecology [31,32] and stock discrimination [28,29,30]. Crespi-abril et al. [33] discovered that the shape of the beaks of *Illex argentinus* did not vary between cohorts and sexes nor between different ontogenetic phases. Jones et al. [19] confirmed that Patagonian squid (*Doryteuthis gahi*) populations had complex structures and high intra-species variations in body shape. Sexual dimorphism of the beak is a common phenomenon in some cephalopods [15,34,35]. However, the sexual dimorphism of the beak in some cephalopods at different ontogenetic stages is unknown, and little is known about the inter-population variation in beak shapes of Cephalopods species.

Cephalopods are diverse in China seas [23,36,37,38]. *O. minor*, *U. edulis*, *S. esculenta*, and *Sthenoteuthis oualaniensis*, representatives of Octopodiformes and Decapodiformes in the subclass Coleoidea, are important economic cephalopod species in coastal waters of China [39,40,41]. This study aims to assess variation in the shape of beaks in different ontogenetic stages of these four squid species and to analyze the sex-linked differences in beak shape and size, relating these differences to relevant factors in the life history of these species. Our analysis will expand the current knowledge about the beak development pattern of cephalopods.

## 2. Materials and Methods

### 2.1. Specimens

The samples were collected from 2018 to 2021 by Chinese commercial jigging and trawl vessels in the East China Sea and South China Sea and included 198 *O. minor*, 205 *U. edulis*, 198 *S. esculenta*, and 218 *Sthenoteuthis oualaniensis* (Table 1). All samples were immediately frozen to −18 °C and shipped back to the laboratory for biological experiments.

Dorsal mantle length (ML) of each specimen was measured by measuring tape and the sex and gonadal maturity of each species were then determined [42,43,44]. In this study, two ontogenetic stages were considered, catalogued as mature and immature according to the maturity stages proposed for each taxon (*O. minor* and *S. esculenta*: [42,44]; *U. edulis* and *Sthenoteuthis oualaniensis*: [43]). After completing biological experiments as above, the beaks were removed from the buccal mass with tweezers, cleaned, placed in a glass bottle containing 75% ethyl alcohol, and numbered for subsequent analysis.

### 2.2. Data Acquisition

**Figure 1:**
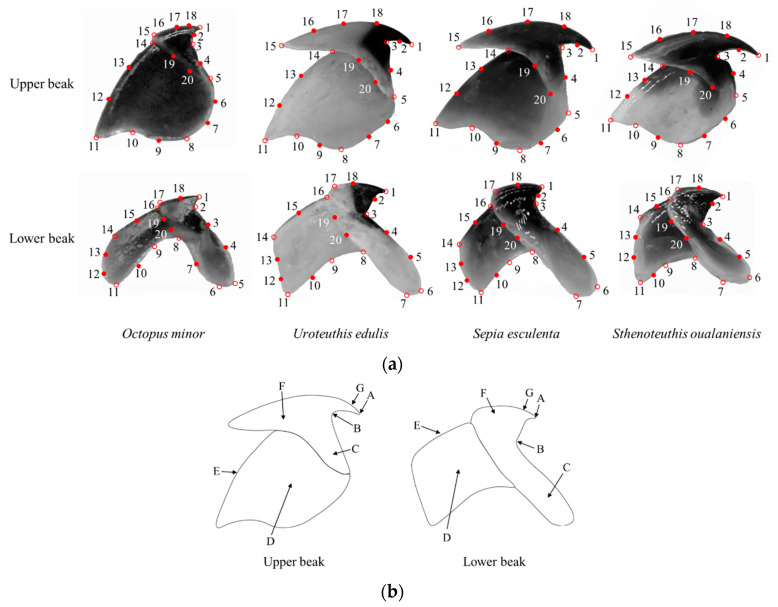
Shape and digitized landmarks (hollow dots) and semi-landmarks (solid dots) of the Cephalopods beak. (**a**): Landmark positions. (**b**): Shape description—A: Rostral tip, B: Jaw angle, C: Wing, D: Lateral wall, E: Crest, F: Hood, G: Rostrum.

We focused on lateral profiles of the upper and lower beaks (Figure 1), which were commonly used in previous studies [15,29,45]. The beak photos were taken in a small photography shed. After debugging the small photography shed and placing the scale ruler, the NiKonD750 camera was fixed with a tripod (the parameters of shooting lens are Micro 105 mm f/2.8). To prevent shooting errors, the positions of beaks, camera and focusing point were kept unchanged during shooting. Beak samples were taken out one by one from the glass bottle and positioned, photos were taken immediately. In order to facilitate reading image data with the software and ensure the efficiency of subsequent shape analysis, the images were preprocessed in Photoshop CS6. The obtained images were then used to establish landmarks.

The images of beaks were each named according to species, sex, ontogenetic stage, and the specimen number. We added semilandmarks to better represent beak shape [46]. According to the previous studies [15], 8 landmarks and 12 semilandmarks were marked on the upper beak (Figure 1). Additionally, 10 landmarks and 10 semilandmarks were marked on the lower beak. Landmarks and semilandmarks of the beaks were digitized in 2D with the software TpsDig2 [47]. The coordinate data were read with the “readland.tps” function in the R package “geomorph” [48]. The landmarks and semilandmarks were established with the “define.sliders” function in the R package “geomorph” [48]. The process of establishing landmarks was repeated twice to minimize measurement errors [49]. The two coordinate replicates differed by less than 5%; the data were averaged for the subsequent shape analysis.

### 2.3. Geometric Morphometrics Analysis

The ontogenetic variation and sexual dimorphism of beak shape of *O. minor*, *U. edulis*, *S. esculenta*, and *Sthenoteuthis oualaniensis* were analyzed with GM methods.

All morphological measurements and statistical analyses were performed in the R 4.0.5 “geomorph” package [48]. Firstly, the landmarks of all samples were transformed and rotated in order to eliminate the effects of non-shape variation by generalized Procrustes analysis (GPA) with the “gpagen” function [8,48]. The squared sum of the distances from all landmarks to the centroid was defined as the centroid size, which can be used to represent the size of the organism’s structure [50]. Therefore, the centroid size obtained from the landmarks data of beaks was used as an index of the beak size in order to compare the variation in the beak size between immature and mature specimens. We conducted a Procrustes analysis of variance (ANOVA) with the “procD.lm” function to assess ontogenetic differences and sexual dimorphism in the upper and lower beak shape [48,51]. In order to reduce the spatial dimensions of shape data, principal component analysis (PCA) of landmarks data was performed with the “gm.prcomp” function to determine the main components of the shape variation in the upper and lower beak samples [48], and the beak shape variation associated with principal component 1, 2 (PC1, PC2) and principal component 3, 4 (PC3, PC4) was graphically depicted. Then, we also performed the multivariate regression analysis to compare the allometric patterns of beaks in ontogenetic stages based on the relationship between beak shape and centroid size [2,10]. Finally, the shape variation of beaks by ontogenetic stage was visualized with thin-plate spline (TPS) deformation grids of the “plotRefToTarget” function [48,52].

## 3. Results

### 3.1. Ontogenetic Variation

**Figure 2:**
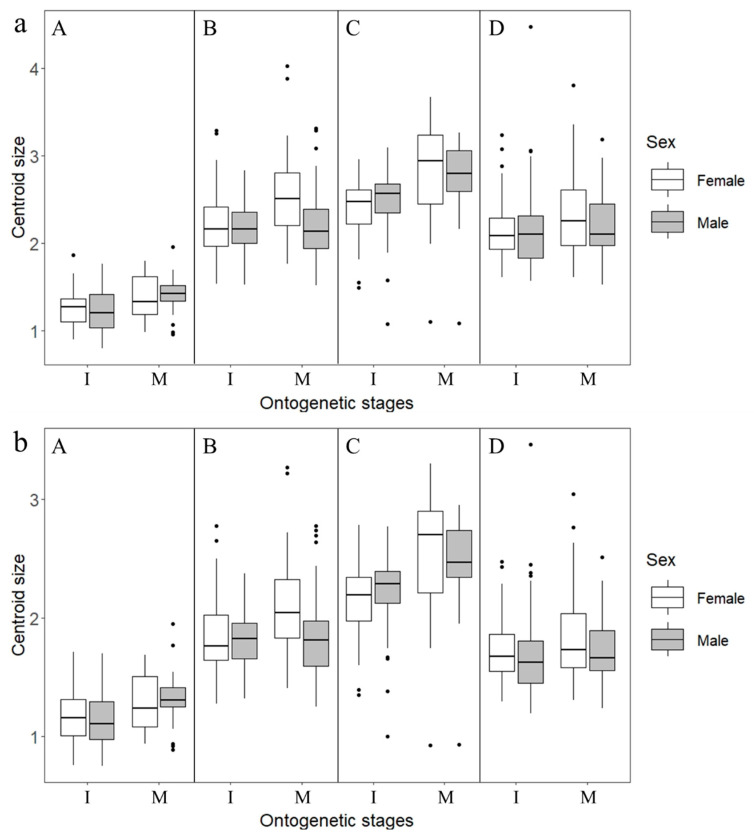
Beak centroid size variation of four cephalopods in different ontogenetic stages. (**a**): Upper beak; (**b**): Lower beak. I: Immature stages; M: Mature stages; A: *Octopus minor*; B: *Uroteuthis edulis*; C: *Sepia esculenta*; D: *Sthenoteuthis oualaniensis*.

There are significant differences in the size of the upper beaks among the four cephalopod species (*p* < 0.05; Table 2), *S. esculenta* had the largest beaks, followed by *U. edulis* and *Sthenoteuthis oualaniensis*. The beaks of *O. minor* were the smallest. The upper beaks are slightly larger than the lower beaks in all four species (Figure 2). In addition, during ontogenesis, beak size also increased accordingly. The beak size of *O. minor* and *S. esculenta* showed the more significant variation (Figure 2a,c) compared to those of *U. edulis* and *Sthenoteuthis oualaniensis* between ontogenetic stages, respectively (Figure 2b,d).

**Figure 3:**
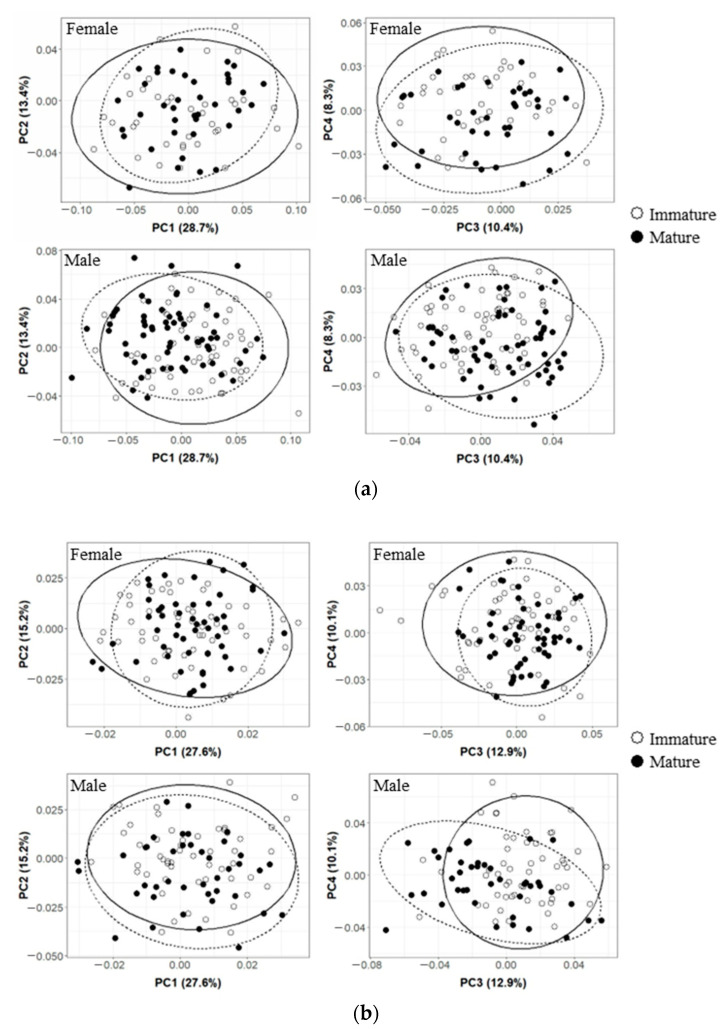

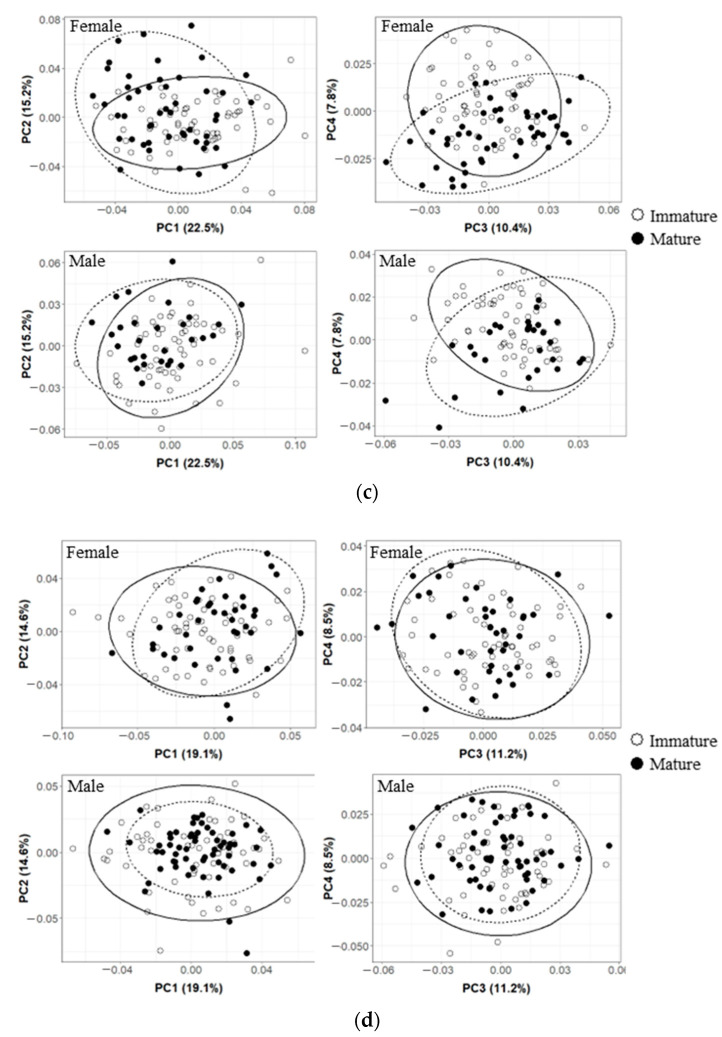
Results of principal component analysis (PCA) of upper beak of the four cephalopods, showing the first principal component (PC1) versus (PC2) and (PC3) versus (PC4) shape variation with 95% ellipse confidence intervals of immature (solid line) and mature (dashed line) beaks. (**a**) *Octopus minor*. (**b**) *Uroteuthis edulis*. (**c**) *Sepia esculenta*. (**d**) *Sthenoteuthis oualaniensis*.

**Figure 4:**
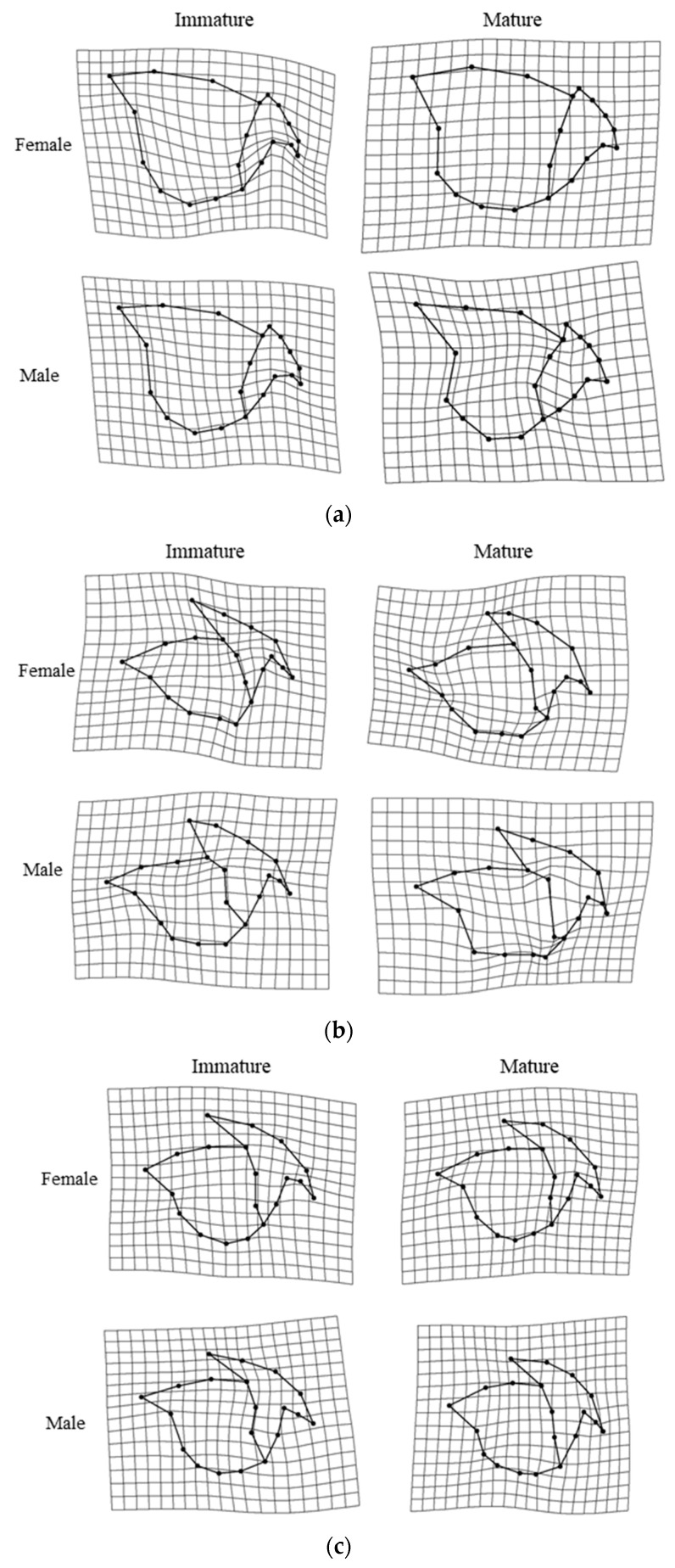

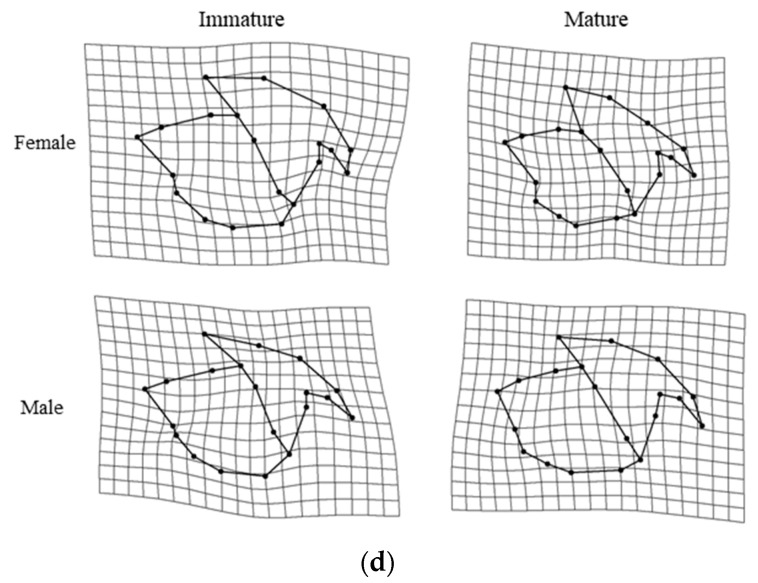
Thin-plate spline deformation grids of upper beak of the four cephalopods considered in different ontogenetic stages. (**a**) *Octopus minor*. (**b**) *Uroteuthis edulis*. (**c**) *Sepia esculenta*. (**d**) *Sthenoteuthis oualaniensis*.

**Figure 5:**
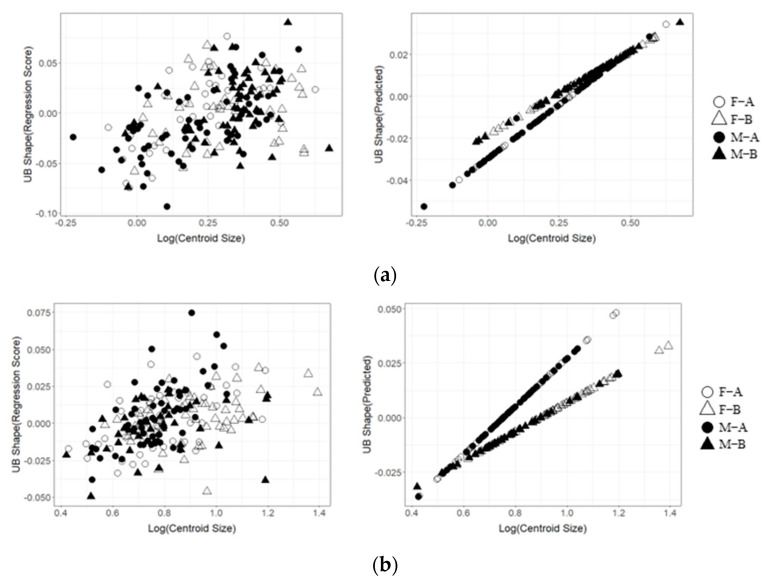

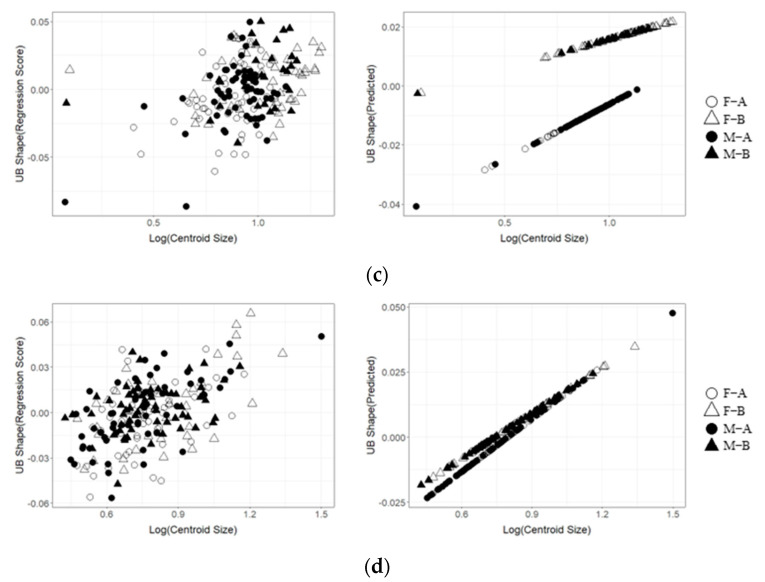
Correlativity of the regression scores of upper beak shape and predicted values versus log(Centroid Size). F–A: Immature, female; F–B: Mature, female; M–A: Immature, male; M–B: Mature, male. (**a**) *Octopus minor*. (**b**) *Uroteuthis edulis*. (**c**) *Sepia esculenta*. (**d**) *Sthenoteuthis oualaniensis*.

The results of the Procrustes analysis of variance showed that the shapes of the upper beaks of *O. minor*, *U. edulis*, and *S. esculenta* differed significantly between ontogenetic stages (*p* < 0.05; Table 2), but the shape of the upper beak of *Sthenoteuthis oualaniensis* showed no significant difference between immature and mature stages (*p* > 0.05; Table 2). Results of principal component analysis also suggest more overlap between the immature and mature stages of the upper beak of the *Sthenoteuthis oualaniensis* (Figure 3d). The results of principal component analysis on the shape of the upper beak indicated that the first four principal components (PC1, PC2, PC3, and PC4) altogether accounted for 60.8%, 65.8%, 55.9% and 53.4% of the variation of the shapes of the upper beaks of *O. minor*, *U. edulis*, *S. esculenta* and *Sthenoteuthis oualaniensis* and the upper beaks between immature and mature stages could be better distinguished in the scatter plot of principal components (Figure 3a–d). The results of the deformation mesh analysis of thin-plate splines deformation grids showed that the main shape variations of the upper beaks of *O. minor*, *U. edulis* and *S. esculenta* between immature and mature stages occurred in the rostrum (Landmarks 1, 2, and 3), hood (Landmarks 16, 18, and 19), wing (Landmarks 4 and 20) and lateral wall (Landmarks 7, 10, 11, and 13) (Figure 4). Although the morphological variations in upper beaks of each species occurred at similar sections between immature and mature stages, four cephalopod species still had different shape variation directions in various parts between immature and mature stages. The rostra of the *O. minor* and *S. esculenta* gradually became shorter, more blunt and thicker (Figure 4a,c), but *U. edulis* became longer, sharper and thinner (Figure 4b). The hoods of *O. minor* and *U. edulis* became more curved and wider (Figure 4a,b), whereas the hoods of *S. esculenta* became curved and compressed (Figure 4c). The lateral wall of the upper beak of each species became wider in different directions. In addition, differences were apparent in changes of upper beak shape with increasing upper beak size (Figure 5a–d) and the predicted shape of the upper beak was positively correlated with the upper beak size (Figure 5a–d).

**Figure 6:**
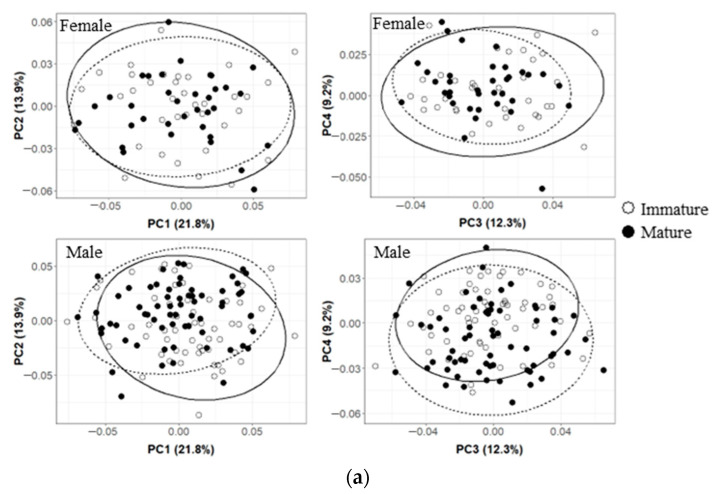

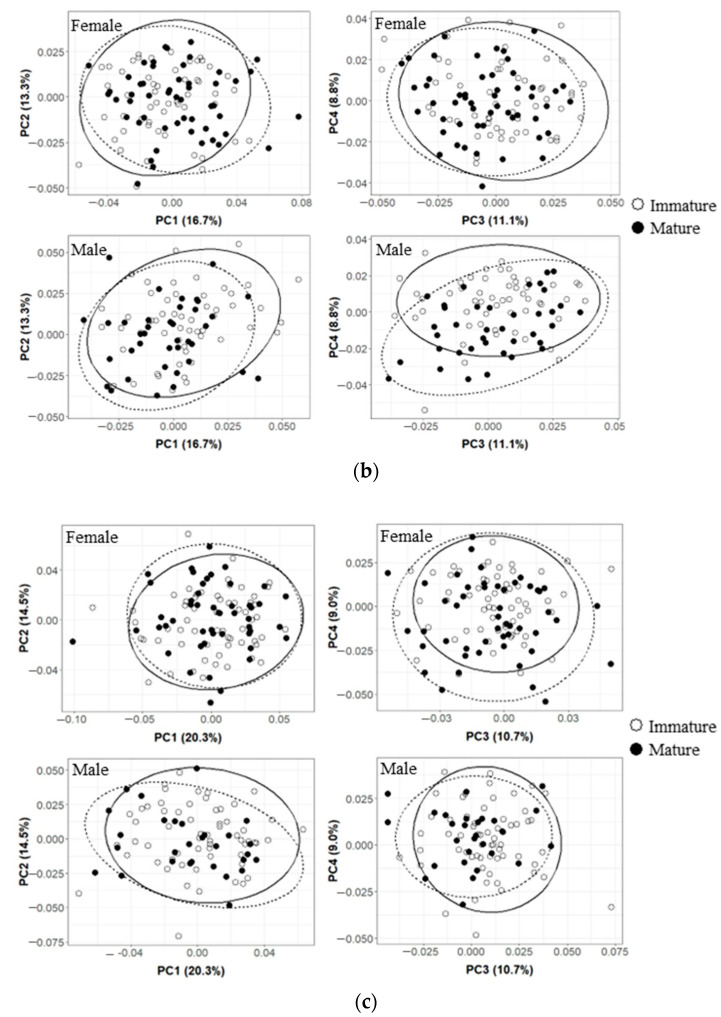

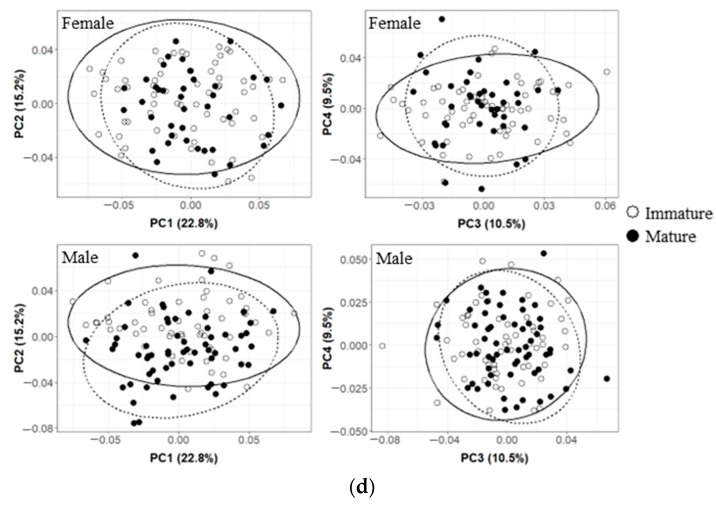
Results of principal component analysis (PCA) of lower beak of the four cephalopods, showing the first principal component (PC1) versus (PC2) and (PC3) versus (PC4) shape variation with 95% ellipse confidence intervals of immature (solid line) and mature (dashed line). (**a**) *Octopus minor*. (**b**) *Uroteuthis edulis*. (**c**) *Sepia esculenta*. (**d**) *Sthenoteuthis oualaniensis*.

**Figure 7:**
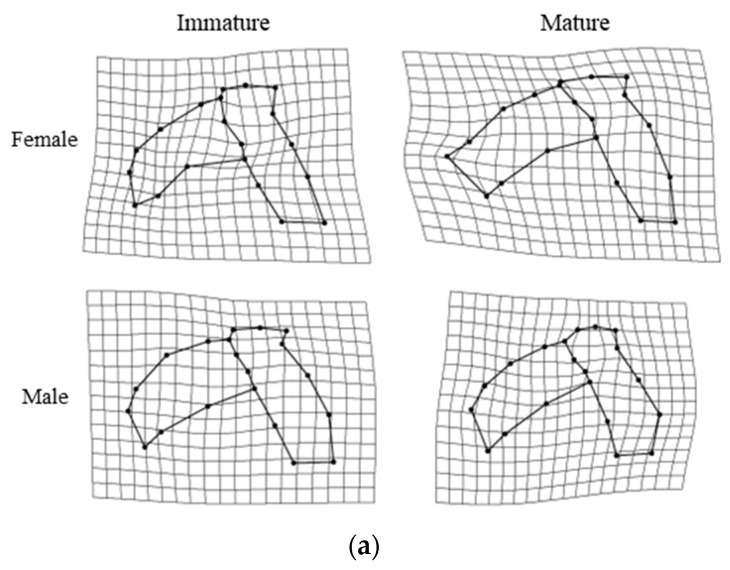

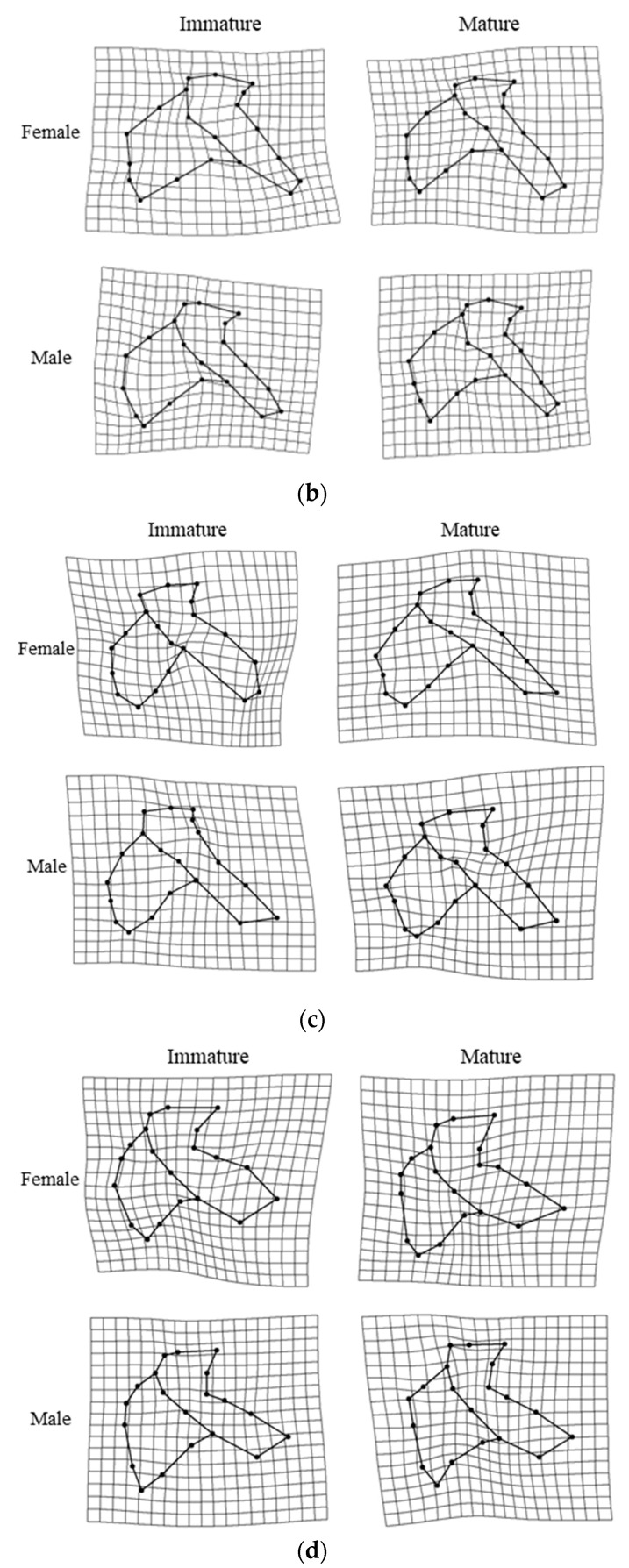
Thin-plate spline deformation grids of lower beak of the four cephalopods considered in different ontogenetic stages. (**a**) *Octopus minor*. (**b**) *Uroteuthis edulis*. (**c**) *Sepia esculenta*. (**d**) *Sthenoteuthis oualaniensis*.

**Figure 8:**
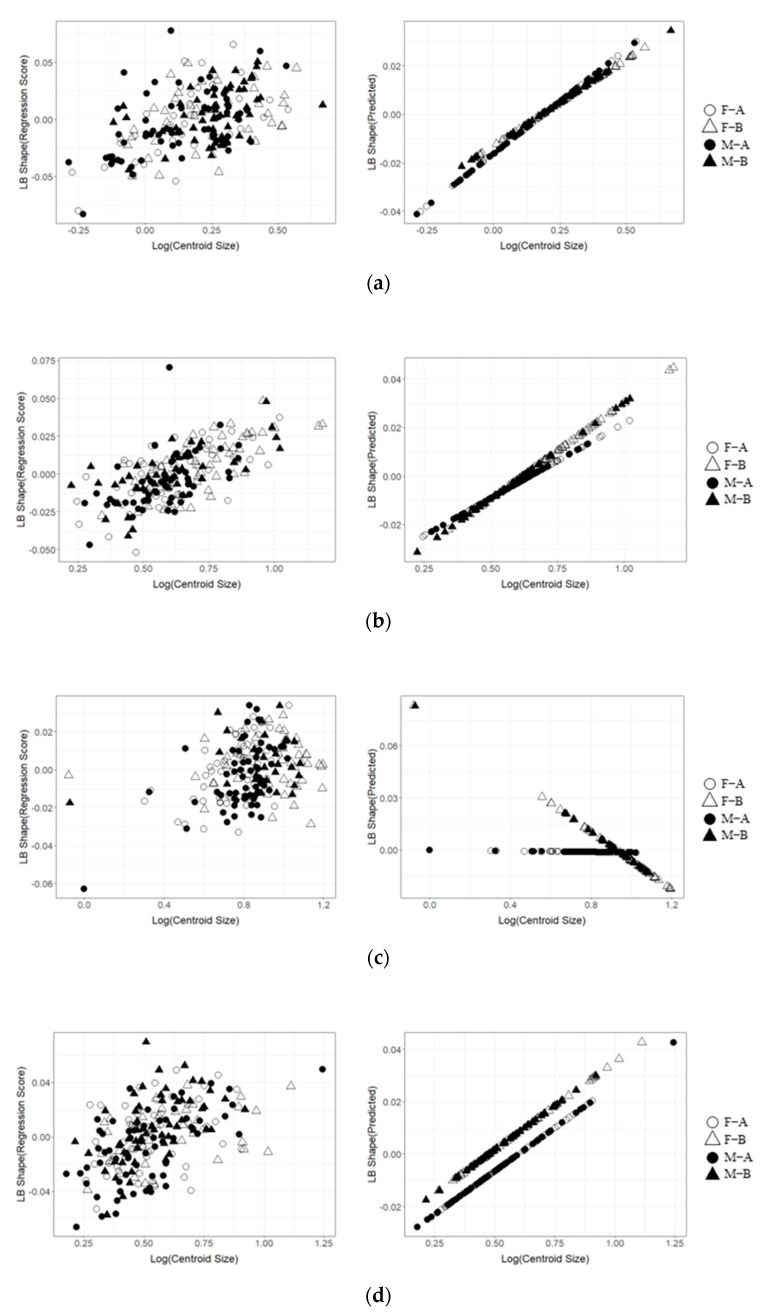
Correlativity of the regression scores of lower beak shape and predicted values versus log(Centroid Size). F–A: Immature, female; F–B: Mature, female; M–A: Immature, male; M–B: Mature, male. (**a**) *Octopus minor*. (**b**) *Uroteuthis edulis*. (**c**) *Sepia esculenta*. (**d**) *Sthenoteuthis oualaniensis*.

The results of the Procrustes analysis of variance showed that the size of the lower beak of four species showed significant variation (*p* < 0.05; Table 3). The shapes of the lower beaks of *U. edulis*, *S. esculenta*, and *Sthenoteuthis oualaniensis* were significantly different between immature and mature stages (*p* < 0.05; Table 3), but the shape of the lower beaks of *O. minor* showed no significant variation (*p* > 0.05; Table 3). The first four principal components (PC1, PC2, PC3, and PC4) altogether accounted for 57.2%, 49.9%, 54.5% and 58.0% of the variation in lower beak shape of the lower beaks of *O. minor*, *U. edulis*, *S. esculenta*, and *Sthenoteuthis oualaniensis* (Figure 6a–d). The explanatory rate of the first 4 principal components for the variation of the lower beak was slightly lower than that for the variation of the upper beak. The scatter plots of principal components for the beak shape in immature stages partially coincided with those in mature stages, but they could still explain the variation in the beak shape among various ontogenetic stages (Figure 6b–d). The results of the deformation mesh analysis of thin-plate splines deformation grids showed that the main variations of the shapes of the lower beaks of *U. edulis*, *S. esculenta*, and *Sthenoteuthis oualaniensis* in the immature and mature stages occurred at rostrum (Landmarks 1 and 18), hood (Landmark 17), wing (Landmarks 4, 5, 6, and 7) and lateral wall (Landmarks 9, 11, and 14) (Figure 7). In mature stages, *U. edulis* had sharper rostrum, wider wing, longer hood, and wider lateral wall (Figure 7b) and similar variations were observed in the lower beaks of *S. esculenta* and *Sthenoteuthis oualaniensis* (Figure 7c,d). In particular, the shapes of the lower beaks of different species showed different variations. For instance, the hoods of *U. edulis* and *S. esculenta* became more curved (Figure 7b,c), whereas the hood of *Sthenoteuthis oualaniensis* flattened (Figure 7d). Moreover, the variation of the shape of the lower beak with size was not significant (Figure 8b–d). Compared with the upper beak, the lower beak showed significant difference in variation rate of shape among four species in both immature and mature stages. The beak shape variation pattern of *S. esculenta* was different from other species. *Sthenoteuthis oualaniensis* had similar variation pattern of the lower beak (Figure 8d) and the predicted shape of the lower beak was positively correlated with beak size. The shape of the lower beak of *S. esculenta* showed no significant variation with the increase of beak size in immature stages, but the shape of the lower beak of *S. esculenta* was negatively correlated with beak size in mature stages (Figure 8c, right).

### 3.2. Sexual Dimorphism

Firstly, the results of the Procrustes analysis of variance indicated that the shapes of the upper beaks of *O. minor*, *U. edulis* and *Sthenoteuthis oualaniensis* were significantly different between female and male (*p* < 0.05; Table 2), whereas the shapes of the upper beaks of *S. esculenta* showed no sexual dimorphism (*p* > 0.05; Table 2). The scatter plot of principal components indicated that the spatial proportions of the upper beaks of female and male individuals showed some differences (Figure 3a,b,d). The upper beak of male individuals of *O. minor* and *U. edulis* had a more protruding lower recess of the lateral wall, wider hood and sharper rostra than did female individuals in immature and mature stages (Figure 4a,d). Unlike *O. minor* and *U. edulis*, the upper beaks of female individuals of *Sthenoteuthis oualaniensis* had sharper and longer rostra than did male individuals (Figure 4b). The multiple regression analysis results showed that the upper beaks of *O. minor* and *Sthenoteuthis oualaniensis* were not significantly different between male and female individuals (Figure 5a,d, left), but the upper beak of *U. edulis* was significantly different between male and female individuals (Figure 5b, left). The shapes of the upper beaks of female and male individuals in different species showed the same variation of shape with increasing size (Figure 5, right).

The results of the Procrustes analysis of variance indicated that the *S. esculenta* showed significant sexual dimorphism in the shape of the lower beak (*p* < 0.05; Table 3). The shapes of the lower beaks of *O. minor*, *U. edulis* and *Sthenoteuthis oualaniensis* were not significantly different between females and males (*p* > 0.05; Table 3). The scatter plot of principal components of *S. esculenta* indicated that the spatial proportions of the principal components of the lower beak shapes were significantly different between female and male individuals (Figure 6c). In immature stages, female individuals of *S. esculenta* had sharper rostra and more curved hoods of the lower beaks, wider wings, and slightly compressed lateral wall than male individuals. In mature stages, male individuals of *S. esculenta* had wider hoods, wings and lateral wall of the lower beak than did female individuals (Figure 7c). Multiple regression analysis showed that the difference in the lower beak between male and female individuals was not significant (Figure 8c, left). The shape of the lower beaks of female and male individuals showed the same variation of shape with increasing size (Figure 8c, right).

## 4. Discussion

### 4.1. Ontogenetic Pattern of Beak Shape

Cephalopods are short-lived species, so their growth and development are closely related to the habitat environment and their feeding habits are different among various ontogenetic stages [53,54,55]. As a feeding organ of cephalopods, the beak is one of the hardest parts of the cephalopod body and mainly used to bite and fix prey [56,57]. In addition, genetic factors also contribute to the shape differentiation of beaks among different species and different ontogenetic stages [58,59]. This phenomenon is possibly the consequence of the adaptive evolution of different species to habitat environment changes. Therefore, the beak shape of different species during ontogeny may be affected by many factors and display different variation patterns.

In this study, the upper and lower beaks of each cephalopod species showed significant changes in the hood, wing, and rostrum between immature and mature individuals (Figure 4 and Figure 7). The beaks in the cephalopod buccal mass are controlled by musculature in the buccal mass, including the anterior mandibular muscle, lateral mandibular muscle, posterior mandibular muscle and superior mandibular muscle [60,61,62]. The parts of the upper and lower beaks are surrounded by these muscles, include the hood, lateral wall, crest and wing [60,61,62,63]. Since the diets of each cephalopod species are gradually expanded to bigger and harder foods as they grow [41,55,64,65,66], larger beaks are required to produce a stronger bite. Therefore, we assumed that the increase in the proportion of the hood, lateral wall, crest and wing of upper and lower beaks relate to muscle growth, insertion and bite cycle and help to improve the bite force of the beaks. However, the variation pattern of different kinds of beaks were slightly different. For instance, the hoods of the lower beaks of *U. edulis* and *S. esculenta* became more curved with growth (Figure 7b,c) and the hoods of the lower beaks of *Sthenoteuthis oualaniensis* became flat (Figure 7d). The habitats of *U. edulis* and *S. esculenta* are more similar (Table 1). Therefore, the different development patterns suggested differences in the function and growth mechanisms of beaks among different cephalopods. These differences may result from adaptation of each species to habitat and feeding preference during ontogeny.

Larger feeding organs and larger beaks specifically would allow organisms to eat larger and harder food [41,55,65,67]. In this study, *O. minor* inhabits the benthic environment of temperate waters and mainly preys on crustaceans [54,64]). *S. esculenta* is also a demersal shallow-water species that preys on microcrustaceans in its alevin stage, and bigger crustaceans in its adult stage [53,65]. Due to the differences in feeding types and the complexity of the benthic habitat at different ontogenetic stages, the rostra of *O. minor* and *S. esculenta* may be subjected to greater abrasion during feeding and gradually become dull in the maturation stage (Figure 4a,c and Figure 7c). After sexual maturity, *U. edulis* in the East China Sea mainly preys on juvenile fish of Scombridae and other fishes [55], which are softer than the foods preyed by *O. minor* and *S. esculenta*. Sharper upper beaks can help to quickly secure prey and facilitate eating prey [57,68]. As a result, the rostra of *O. minor* and *S. esculenta* became dull and the rostrum of *U. edulis* became sharp. *Sthenoteuthis oualaniensis* mainly preys on microcrustacean and crustaceans in paralarval and adult stages, respectively [66]. These mature individuals need to eat more food for more energy to sustain life activities such as growth and reproduction [34]. In addition, usually cephalopod beak growth rate of the immature stage is faster. When the carcass grows to a certain stage, the growth rate of the beaks slows [33,69,70]. Therefore, the results in this study suggested that feeding strategies in different ontogenetic stages might be responsible for the difference of beak shape.

In addition to the differences in the internal formation mechanism of beaks and feeding habits, the habitat environment may also affect the variation of beaks. Firstly, the habitat environments of different cephalopod species are different. *O. minor* are benthic cephalopod species [53,64,65], whereas *U. edulis* and *S. esculenta* inhabit the demersal, and *Sthenoteuthis oualaniensis* inhabits the pelagic [71,72,73]. Secondly, in the growth process of a single species, due to migration and other living habits, the habitat environment is different in various ontogenetic stages. The East China Sea and South China Sea are greatly affected by the continental coastal currents, black tides and southwest and northeast monsoons, and the climate is complex and variable. The four species had a wide swimming range and variable habitat environment in the growth process [55]. Roscian et al.’s [68] study highlights the importance of habitat as a driver of variation in beak shape. The short, blunt, thick rostra of *O. minor* and *S. esculenta* are likely more useful for breaking shells (Figure 4a,c), while the long, sharp and thin rostra of *U. edulis* and *Sthenoteuthis oualaniensis* are likely more efficient at piercing and tearing fish (Figure 7a,c). Long and extremely mobile arms of benthic cephalopods allow them to capture, maintain and manipulate prey without needing to kill it rapidly. In contrast, pelagic cephalopod hunt by projecting the tentacles and rapidly bringing back the prey toward the beak to kill it [54,57,64,68]. Therefore, beak shape changes between immature and mature may reflect an adaptation of cephalopod species to the habitats and diet resources.

### 4.2. Sexual Dimorphism of Beak Shape

Gender selection, ecological differences, etc. may cause the differences in size and shape between female and male individuals and the shape differences of biological individuals between female and male individuals are called sexual dimorphism [17,18]. Some studies confirmed the presence of sexual dimorphism in cephalopod beaks [15,35]. In this study, the upper beaks of female individuals of each cephalopod species were larger than those of male individuals in the mature stages (*p* < 0.05) (Figure 2; Table 2 and Table 3). Female squids always grow faster than males during ontogeny, which ultimately leads to the subtle sexual dimorphism [34]. We also suggested that the shapes of the upper beaks of *O. minor* and *U. edulis* and the shape of the lower beak of *S. esculenta* showed gender differentiation in various ontogenetic stages (Table 2). The upper beak of *O. minor* and *Sthenoteuthis oualaniensis* females had a more protruding crest and wider hood than that of males (Figure 4a,d). In the mature stages, the hood and wing of the beaks of females of *S. esculenta* were wider than those of males (Figure 4d and Figure 7d). Similarly, the beak shapes of neon flying squid (*Ommastrephes bartramii*) also showed significant difference between females and males [15]. The female beaks, with larger, wider hood and sharp rostra, also might be related to the high quality of food requirements after sexual maturation since larger beaks provide stronger feeding ability to prey on high-level food. This also may reflect that beak shape changes in different sexes are an adaptation of cephalopod species to the habitats and diet resources.

### 4.3. Variations in the Size and Shape of Beaks

Allometry has long been an important focus of the study on evolution and growth and GM is a flexible and powerful tool for studying shape structure evolution and development [12,74]. The multiple regression analysis in this study revealed some differences in allometric variation of beaks of each species (Figure 5 and Figure 8). The intensity of feeding in some cephalopods varied significantly with the growth stage [55,64,66]. The intensity of feeding of individuals gradually increased in the immature stages and decreased after maturity was reached; some individuals did not eat at all after spawning [75]. In this study, the upper beaks of *O. minor*, *U. edulis*, and *S. esculenta* show faster variation rates of shape in their immature stages (Figure 5). It was inferred that the different feeding demands in various ontogenetic stages affected the growth rate and shape on their upper beaks, so beaks grow faster in immature stages. In addition, the rostrum is the main part for cutting foods [56,57]. Therefore, the variation of rostrum of each species was bigger, as indicated in thin-plate spline deformation grids analysis (Figure 4 and Figure 7). Hence, the phenomenon of allometry in the beak shape occurred. In this study, we found that the upper beaks had a higher variation rate of shape than the lower beaks (Figure 5b,c and Figure 8b,c). It was shown that the upper beak grew faster compared to the lower beak, which could also explain why the upper beaks of the four species were slightly larger than the lower beaks (Figure 2). In some cephalopod species, the upper beak grew faster than the lower beak [69,76]. Similar differences in other cephalopods were ascribed to the earlier formation of the upper beak during embryonic development [77]. This study suggests that this difference of size on the upper and lower beaks is also related to differences in allometry.

## 5. Conclusions

Through geometric morphometrics analysis, we found that the beaks of *O. minor*, *U. edulis*, *S. esculenta*, and *Sthenoteuthis oualaniensis* showed different variation patterns, displaying sexual dimorphism and allometry between ontogenetic stages. Habitat difference (diet difference and intra and interspecific competition) may drive ontogenetic variation in beak shape. This study has furthered our understanding of the beak development pattern among four cephalopods species. The detection of additional sexual differences and their expression during ontogeny in other cephalopod species is also highly interesting and necessary, considering the strongly specialized life history in cephalopods, and different habitats.

## Figures and Tables

**Table 1:** Sample information of four cephalopods in this study.

Species	Ontogenetic Stages	Quantity of Samples (F, M)	Mantle Length (mm) (F, M)
*Octopus minor*	Immature	97 (37, 60)	16–69, 12–74
	Mature	102 (39, 63)	20–72, 20–79
*Uroteuthis edulis*	Immature	110 (55, 55)	92–190, 86–182
	Mature	95 (53, 42)	103–240, 100–232
*Sepia esculenta*	Immature	120 (60, 60)	51–129, 56–139
	Mature	78 (49, 29)	62–160, 63–154
*Sthenoteuthis oualaniensis*	Immature	118 (60, 58)	101–171, 100–189
	Mature	100 (60, 40)	104–296, 109–187

**Table 2:** Procrustes ANOVA of the shapes of upper beaks of the four cephalopods considered in different ontogenetic stages.

*Octopus minor*	*df*	SS	MS	*F*	*Z*	*p*
Size	1	0.0556	0.0556	10.9116	5.0287	**0.001**
Ontogenetic stages	1	0.0109	0.0109	2.1395	1.8142	**0.035**
Sex	1	0.0160	0.0160	3.1475	2.4676	**0.009**
Size × ontogenetic stages	1	0.0117	0.0117	2.2959	2.0383	**0.024**
Size × sex	1	0.0043	0.0043	0.8494	−0.1484	0.549
Ontogenetic stages × sex	1	0.0079	0.0079	1.5491	1.1651	0.125
Size × ontogenetic stages × sex	1	0.0052	0.0052	1.0255	0.2676	0.385
Residuals	191	0.9726	0.0051			
Total	198	1.0843				
*Uroteuthis edulis*	*df*	SS	MS	*F*	*Z*	*p*
Size	1	0.0335	0.0335	6.3105	3.4049	**0.001**
Ontogenetic stages	1	0.0134	0.0134	2.5257	2.4851	**0.007**
Sex	1	0.0103	0.0103	1.9345	1.9292	**0.029**
Size × ontogenetic stages	1	0.0108	0.0108	2.0415	1.5520	0.066
Size × sex	1	0.0159	0.0159	3.0031	2.1099	**0.021**
Ontogenetic stages × sex	1	0.0126	0.0126	2.3824	2.3226	**0.012**
Size × ontogenetic stages × sex	1	0.0127	0.0127	2.3879	1.8585	**0.030**
Residuals	197	1.0455	0.0053			
Total	204	1.1548				
*Sepia esculenta*	*df*	SS	MS	*F*	*Z*	*p*
Size	1	0.0286	0.0286	8.3690	4.9628	**0.001**
Ontogenetic stages	1	0.0252	0.0251	7.3539	5.1175	**0.001**
Sex	1	0.0047	0.0047	1.3757	0.9843	0.172
Size × ontogenetic stages	1	0.0108	0.0108	3.1603	2.8167	**0.003**
Size × sex	1	0.0069	0.0069	2.0157	1.8508	**0.038**
Ontogenetic stages × sex	1	0.0019	0.0019	0.5642	−1.1075	0.874
Size × ontogenetic stages × sex	1	0.0026	0.0026	0.7575	−0.4160	0.661
Residuals	190	0.6497	0.0034			
Total	197	0.7304				
*Sthenoteuthis oualaniensis*	*df*	SS	MS	*F*	*Z*	*p*
Size	1	0.0295	0.0295	8.9339	5.4394	**0.001**
Ontogenetic stages	1	0.0037	0.0037	1.1051	0.4886	0.318
Sex	1	0.0066	0.0066	1.9938	2.0165	**0.021**
Size × ontogenetic stages	1	0.0025	0.0024	0.7398	−0.5531	0.698
Size × sex	1	0.0044	0.0044	1.3345	0.8517	0.190
Ontogenetic stages × sex	1	0.0048	0.0048	1.4396	1.1014	0.134
Size × ontogenetic stages × sex	1	0.0055	0.0055	1.6637	1.4738	0.068
Residuals	210	0.6941	0.0033			
Total	217	0.7510				

*df*: degrees of freedom; SS: sum of squares; MS: mean squares’ *F*: test statistics; *Z*: effect size. Bold *p* values indicate significant at *α* = 0.05.

**Table 3:** Procrustes ANOVA of the shapes of lower beaks of the four cephalopods considered in different ontogenetic stages.

*Octopus minor*	*df*	SS	MS	*F*	*Z*	*p*
Size	1	0.0399	0.0399	7.5901	5.9824	0.001 *
Ontogenetic stages	1	0.0069	0.0069	1.3162	0.8405	0.193 ^ns^
Sex	1	0.0091	0.0091	1.7232	1.5217	0.064 ^ns^
Size × ontogenetic stages	1	0.0066	0.0066	1.2474	0.7334	0.235 ^ns^
Size × sex	1	0.0064	0.0064	1.2192	0.7083	0.244 ^ns^
Ontogenetic stages × sex	1	0.0082	0.0082	1.5549	1.2435	0.114 ^ns^
Size × ontogenetic stages × sex	1	0.0066	0.0066	1.2623	0.7695	0.219 ^ns^
Residuals	192	1.0084	0.0053			
Total	199	1.0919				
*Uroteuthis edulis*	*df*	SS	MS	*F*	*Z*	*p*
Size	1	0.0350	0.0350	10.1427	6.4009	0.001 *
Ontogenetic stages	1	0.0070	0.0070	2.0207	1.9704	0.025 *
Sex	1	0.0051	0.0051	1.4765	1.1945	0.133 ^ns^
Size × ontogenetic stages	1	0.0043	0.0043	1.2519	0.7994	0.210 ^ns^
Size × sex	1	0.0032	0.0032	0.9257	−0.0621	0.523 ^ns^
Ontogenetic stages × sex	1	0.0084	0.0084	2.4245	2.4116	0.008 *
Size × ontogenetic stages × sex	1	0.0063	0.0062	1.8112	1.7396	0.039 *
Residuals	197	0.6793	0.0034			
Total	204	0.7484				
*Sepia esculenta*	*df*	SS	MS	*F*	*Z*	*p*
Size	1	0.0196	0.0196	5.2965	4.2139	0.001 *
Ontogenetic stages	1	0.0131	0.0131	3.5309	3.3474	0.001 *
Sex	1	0.0094	0.0093	2.5219	2.2754	0.011 *
Size × ontogenetic stages	1	0.0146	0.0146	3.9279	3.3226	0.001 *
Size × sex	1	0.0043	0.0042	1.1461	0.5342	0.305 ^ns^
Ontogenetic stages × sex	1	0.0042	0.0042	1.1229	0.4810	0.320 ^ns^
Size × ontogenetic stages × sex	1	0.0075	0.0075	2.0309	1.7868	0.030 *
Residuals	190	0.7044	0.0037			
Total	197	0.7770				
*Sthenoteuthis oualaniensis*	*df*	SS	MS	*F*	*Z*	*p*
Size	1	0.0315	0.0315	6.7091	4.5043	0.001 *
Ontogenetic stages	1	0.0111	0.0111	2.3571	2.0559	0.017 *
Sex	1	0.0043	0.0043	0.9091	−0.0141	0.502 ^ns^
Size × ontogenetic stages	1	0.0047	0.0047	0.9914	0.1859	0.435 ^ns^
Size × sex	1	0.0151	0.0151	3.2157	2.7576	0.002 *
Ontogenetic stages × sex	1	0.0103	0.0102	2.1853	2.0485	0.024 *
Size × ontogenetic stages × sex	1	0.0060	0.0060	1.2825	0.7839	0.226 ^ns^
Residuals	210	0.9850	0.0047			
Total	217	1.0677				

*df*: degrees of freedom, SS: sum of squares, MS: mean squares, *F*: test statistics, *Z*: effect size, ns: not significant. * *p*: significant at *α* = 0.05.

## Data Availability

The data that support the findings of this study are available from the corresponding author upon reasonable request.
